# Alpha herpesvirus exocytosis from neuron cell bodies uses constitutive secretory mechanisms, and egress and spread from axons is independent of neuronal firing activity

**DOI:** 10.1371/journal.ppat.1012139

**Published:** 2024-04-05

**Authors:** Anthony E. Ambrosini, Kayla M. Borg, Nikhil Deshmukh, Michael J. Berry, Lynn W. Enquist, Ian B. Hogue

**Affiliations:** 1 Department of Molecular Biology, and Princeton Neuroscience Institute, Princeton University, Princeton, New Jersey, United States of America; 2 ASU-Banner Neurodegenerative Research Center, Biodesign Institute, Arizona State University, Tempe, Arizona, United States of America; 3 School of Life Sciences, Arizona State University, Tempe, Arizona, United States of America; Leibniz Institute of Virology (LIV), GERMANY

## Abstract

Alpha herpesviruses naturally infect the peripheral nervous system, and can spread to the central nervous system, causing severe debilitating or deadly disease. Because alpha herpesviruses spread along synaptic circuits, and infected neurons exhibit altered electrophysiology and increased spontaneous activity, we hypothesized that alpha herpesviruses use activity-dependent synaptic vesicle-like regulated secretory mechanisms for egress and spread from neurons. Using live-cell fluorescence microscopy, we show that Pseudorabies Virus (PRV) particles use the constitutive Rab6 post-Golgi secretory pathway to exit from the cell body of primary neurons, independent of local calcium signaling. Some PRV particles colocalize with Rab6 in the proximal axon, but we did not detect colocalization/co-transport in the distal axon. Thus, the specific secretory mechanisms used for viral egress from axons remains unclear. To address the role of neuronal activity more generally, we used a compartmentalized neuron culture system to measure the egress and spread of PRV from axons, and pharmacological and optogenetics approaches to modulate neuronal activity. Using tetrodotoxin to silence neuronal activity, we observed no inhibition, and using potassium chloride or optogenetics to elevate neuronal activity, we also show no increase in virus spread from axons. We conclude that PRV egress from neurons uses constitutive secretory mechanisms: generally, activity-independent mechanisms in axons, and specifically, the constitutive Rab6 post-Golgi secretory pathway in cell bodies.

## Author summary

Alpha herpesviruses, including important human pathogens Herpes Simplex Virus 1 and 2, and Varicella-Zoster Virus, and important veterinary and zoonotic pathogen, Pseudorabies Virus (PRV), are among the very few viruses that naturally infect the nervous system. These viruses cause recurrent herpetic and zosteriform lesions, peripheral neuropathies, and deadly or debilitating central nervous system diseases. Many of the molecular and cellular mechanisms of viral egress and spread remain unknown, particularly in the context of specialized neuronal cell biology. Our results show that PRV uses the constitutive secretory pathway. Therefore, therapies targeting peripheral neuropathic symptoms, elevated neuronal activity, and synaptic vesicle secretory mechanisms are unlikely to affect virus spread in the nervous system.

## Introduction

The alpha herpesviruses, including Herpes Simplex Virus 1 and 2 (HSV-1 & -2), Varicella-Zoster Virus (VZV), and Pseudorabies Virus (PRV; suid alphaherpesvirus 1), are among the very few viruses that have evolved to productively exploit the mammalian nervous system. After initial infection of epithelial tissues, alpha herpesviruses efficiently enter and establish life-long latency in sensory and autonomic peripheral nervous system (PNS) neurons. Upon reactivation of viral replication, virus particles undergo anterograde axonal transport, exocytosis, and spread back to peripheral tissues, where they cause characteristic recurrent herpetic or zosteriform lesions. Rarely in natural hosts, but more frequently in young, old, immunocompromised, and non-natural host species, alpha herpesviruses can also spread into the central nervous system (CNS), causing acute neurological disease like herpes encephalitis [1]. It is increasingly apparent that alpha herpesviruses can also spread to the CNS asymptomatically, even in healthy, immunocompetent, natural hosts, which might contribute to the development of chronic neurodegenerative diseases [2].

In humans, HSV and VZV cause a variety of sensory neuropathies, including numbness, tingling, itch, and burning pain. In non-natural hosts (e.g. laboratory rodents and many other domesticated mammals), PRV causes an intense pruritus known as “mad itch” [3]. These neuropathic symptoms are thought to be caused by altered electrophysiology of infected neurons: HSV is reported to downregulate expression and cell-surface localization of ion channels that regulate neuronal excitability [4–9] [4,5]. PRV and some syncytial strains of HSV are also reported to induce synchronized spontaneous neuronal activity *in vitro* and in non-myelinated neurons *in vivo* [6–10]. Relevant neuron types, such as postganglionic autonomic neurons and some types of sensory neurons are non-myelinated *in vivo*. The Enquist laboratory previously showed that two viral membrane proteins are required for elevated spontaneous activity induced by PRV *in vitro* and *in vivo*: 1) membrane glycoprotein gB is part of the core fusion complex that mediates membrane fusion during viral entry, and cell-cell fusion leading to syncytia; 2) membrane protein US9 (together with glycoproteins gE and gI) is required for vesicular transport of PRV particles and glycoproteins into the axon of neurons. These findings lead to a model where viral membrane fusion proteins are transported into the axons of infected neurons, form fusion pores between adjacent non-myelinated axons, and cause the neurons to become electrically coupled. This leads to elevated and synchronous activity propagating through the network [11,12]. It remains unclear whether these alterations in neuronal firing activity contribute to egress of viral particles and spread from infected neurons.

Constitutive secretory mechanisms mediate secretion of extracellular cargoes in all cell types, as well as homeostatic maintenance of lipids and proteins that form the plasma membrane itself. In general, protein cargoes are sorted at the trans-Golgi network (TGN) and recycling/sorting endosomes into discrete secretory vesicles. Rab-family GTPases bind the cytosolic face of intracellular membranes to specify organelle identity and regulate essentially all intracellular membrane trafficking. Rab6 is marker of the Golgi, TGN, and post-Golgi secretory vesicles [13–16]. It is considered one of the *bona fide* Golgi Rab proteins, whereas others, like Rab8 and Rab11 participate in both Golgi and endocytic trafficking [13]. Rab6 and Rab8 have been shown to associate with post-Golgi secretory vesicles to mediate their constitutive exocytosis [12–14]. Rab11 proteins function at the interface between endocytic and secretory pathways to regulate constitutive exocytosis of both post-Golgi secretory vesicles and recycling of endocytic cargoes to the plasma membrane [17,18]. Previously, we have demonstrated that the constitutive secretory Rab proteins, Rab6a, Rab8a, and Rab11a, are associated with exocytosis of PRV particles in non-neuronal cells [19,20], and other studies have implicated Rab6 in HSV-1 and VZV assembly/egress [21,22]. Very recently, two preprint manuscripts also support these findings: one has confirmed that Rab6a functions in PRV egress from non-neuronal cells [23], and another from our group has shown that HSV-1 also uses the Rab6 pathway in non-neuronal cells [24].

In neurons and many other professional secretory cell types, exocytosis of signaling molecules such as neurotransmitters and hormones is regulated by intracellular Ca^2+^. Rab3 proteins are present on synaptic and dense core vesicles in neurons, and are required for Ca^2+^-regulated exocytosis [25]. Rab11 is reported to play divergent roles in constitutive versus Ca^2+^-regulated exocytosis: Rab11 proteins participate in constitutive exocytosis in non-neuronal cells, as described above, but are reported to colocalize with Rab3 on synaptic vesicles on neurons, and mediate Ca^2+^-regulated, not constitutive, exocytosis in neuroendocrine cells [26]. Several reports in the alpha herpesvirus literature suggest an association between Ca^2+^-regulated secretory machinery and assembly/egress in specialized cell types. Rab3a was detected colocalized with HSV-1 tegument proteins and glycoprotein vesicles in the axons of neurons [27], and Rab27a, which colocalizes with Rab3 on Ca^2+^-regulated secretory vesicles, was reported to be required for efficient HSV-1 replication in an oligodendrocyte cell line [28].

It remains unclear what secretory mechanisms alpha herpesviruses use to exit from neurons, and whether elevated neuronal activity may promote more efficient viral egress and spread. To address this gap in knowledge, we used live-cell fluorescence microscopy to investigate the secretory mechanisms involved in PRV exocytosis from the cell body, and axonal sorting/transport. In addition, we took advantage of a compartmentalized neuronal culture system to measure egress and spread of PRV from axons, together with pharmacological and optogenetic approaches to modulate neuronal activity. In the cell body, PRV particles colocalize and co-transport with Rab6a, and undergo exocytosis from secretory vesicles marked by Rab6a and Rab8a, indicating that PRV uses the constitutive secretory pathway to exit from the cell body. While Rab6a vesicles are readily detected in axons, and some PRV particles colocalize with Rab6a in the proximal axon, and Rab6a was not detectable on PRV particles in distal axons. Nevertheless, PRV exocytosis from the cell body, and egress and spread from axons was independent of neuronal activity, indicating that PRV uses constitutive secretory mechanisms in both the cell body and axons.

## Results

### Rab6a-positive organelles mediate intracellular transport of PRV particles in the cell body

To investigate the role of Rab6a in the intracellular transport of PRV particles in neurons, we constructed a recombinant virus, PRV IH222, that expresses EmGFP-Rab6a and a red fluorescent (mCherry-VP26) capsid tag. To investigate the PRV assembly and egress pathway in biologically-relevant primary PNS neurons, we prepared dissociated cultures of rat embryonic superior cervical ganglion (SCG) neurons. After 10–14 days in vitro, we infected these SCG neurons with PRV IH222, and imaged by widefield and oblique fluorescence microscopy.

**Fig 1:**
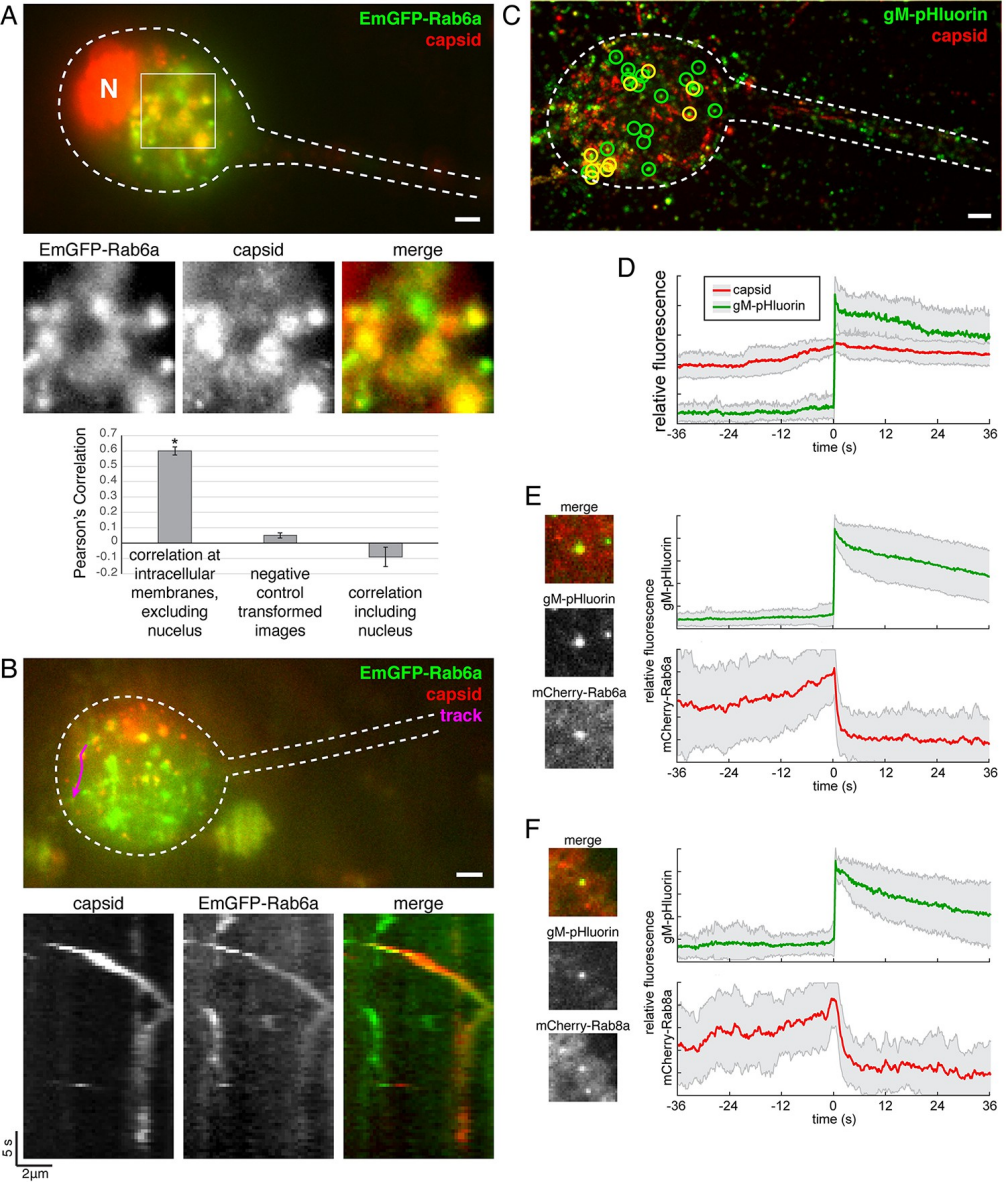
PRV particles use constitutive secretory pathway to exit from the cell body of primary SCG neurons. (A) PRV particles (mCherry-VP26 capsid tag) colocalize with EmGFP-Rab6a in the cell body of SCG neurons. Colocalization quantified from 40 cells over 5 independent experiments. Pearson’s correlation between red and green channels was significantly greater (p<0.01, student’s t-test) at intracellular membranes compared to control conditions. Scale bar represents 4 μm. (B) PRV particles and EmGFP-Rab6a co-transport in the cell body. Kymograph represents particle movement along the indicated track (magenta). Data are representative of >56 cells over 7 independent experiments. Scale bar represents 4 μm. (C) Virus particle exocytosis from neuron cell bodies occurs as early as 5 hpi. Image is a maximum difference projection over a 5.3 min time course. Exocytosis of gM-pHluorin particles that do not contain capsids (green circles) and particles containing both gM-pHluorin and capsids (yellow circles) are indicated. Scale bar represents 4 μm. (D) Ensemble average of gM-pHluorin fluorescence (green line) and mRFP capsid (red line) over 26 exocytosis events. Shaded area represents standard deviation. (E-F) SCG neurons were transduced to express mCherry-Rab proteins, infected with PRV expressing gM-pHluorin, and imaged beginning at 5 hr after PRV infection. Images show representative exocytosis events at the moment of exocytosis (time = 0). Each image is 5 μm square. Line plots are ensemble averages of gM-pHluorin (top, green line) and mCherry-Rab protein (bottom, red line) relative fluorescence. Shaded area represents standard deviation. (E) Rab6a is associated with virus particle exocytosis from neuron cell bodies. Data represent 72 exocytosis events. (F) Rab8a is associated with virus particle exocytosis from neuron cell bodies. Data represent 35 exocytosis events.

**Fig 2:**
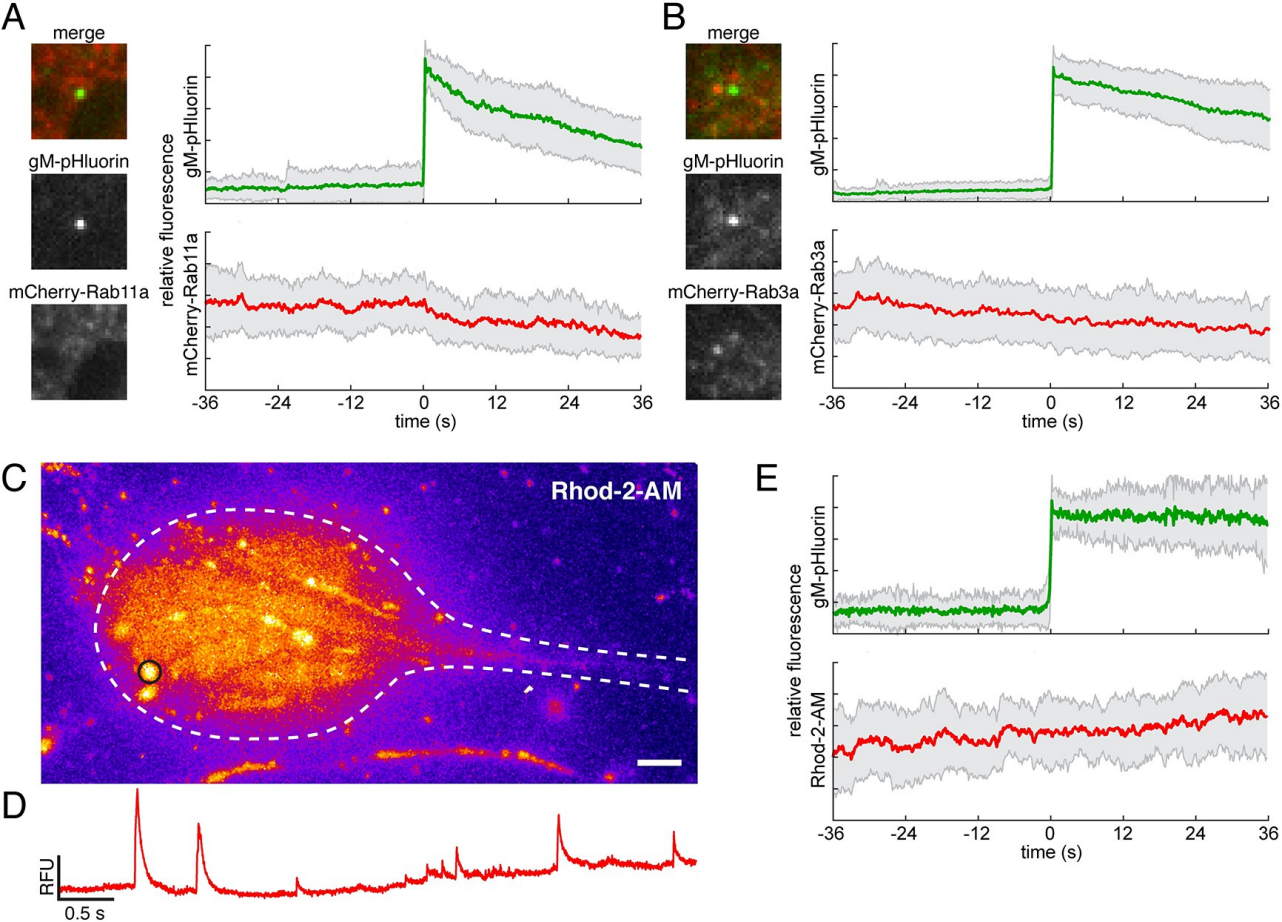
PRV particles exocytosis is not associated with Rab11a, Rab3a, nor local Ca^2+^ signaling. (A-B) SCG neurons were transduced to express mCherry-Rab proteins, infected with PRV expressing gM-pHluorin, and imaged beginning at 5 hr after PRV infection. Images show representative exocytosis events at the moment of exocytosis (time = 0). Each image is 5 μm square. Line plots are ensemble averages of gM-pHluorin (top, green line) and mCherry-Rab protein (bottom, red line) relative fluorescence. Shaded area represents standard deviation. (A) Rab11a is not associated with virus particle exocytosis in neurons. Data represent 41 exocytosis events. (B) Rab3a is not associated with virus particle exocytosis in neurons. Data represent 64 exocytosis events. (C-E) Neurons were infected with PRV expressing gM-pHluorin, loaded with Ca^2+^ sensitive fluorescent dye Rhod-2-AM, and imaged beginning at 5 hpi. (C) Image is a maximum intensity projection of Rhod-2-AM fluorescence over a 6 min time course. Scale bar represents 4 μm. (D) Rhod-2-AM fluorescence intensity at the region of interest indicated in panel C (black circle) over 6 min time course. (E) Virus particle exocytosis in neurons is not associated with local Ca^2+^ transients. Ensemble average of gM-pHluorin (top, green line) and Rhod-2-AM (bottom, red line) relative fluorescence over 27 exocytosis events. Shaded area represents standard deviation.

As has been previously described by many others, using a variety of Golgi markers, Golgi membranes form a juxtanuclear structure, reminiscent of the HCMV assembly compartment, in PRV and HSV-1 infected neurons (e.g. [29,30]). In PRV IH222-infected SCG neurons, we observe strong colocalization between mCherry-VP26 PRV capsids and these EmGFP-Rab6a positive organelles (Fig 1A), which may represent sites of secondary envelopment at the Golgi. We also readily detect fast co-transport of PRV particles with EmGFP-Rab6a vesicles moving towards the cell periphery (Fig 2B).

### PRV particle exocytosis from neuronal cell bodies occurs as early as 5 hours post-infection

To directly measure PRV egress, we previously developed a live-cell fluorescence microscopy assay of virus particle exocytosis [19,20]. We genetically fused pHluorin, a pH-sensitive variant of EGFP, to the first extracellular loop of viral glycoprotein M (gM-pHluorin). When gM-pHluorin is incorporated into virus particles and surrounding secretory vesicle membrane during secondary envelopment, pHluorin fluorescence is strongly quenched in the acidic lumen of the secretory organelle. Upon exocytosis, the pHluorin moiety is exposed to the higher extracellular pH and becomes brightly fluorescent, revealing the location and moment of virus particle egress [19,20].

After 10–14 days in vitro, we infected SCG neurons with PRV 483, expressing gM-pHluorin and a red fluorescent (mRFP-VP26) capsid tag, and imaged by Total Internal Reflection Fluorescence (TIRF) microscopy. In agreement with previous work in non-neuronal cells, we detected exocytosis of individual virus particles based on the sharp increase in gM-pHluorin fluorescence. We detected exocytosis of both virions, categorized based on the colocalization of gM-pHluorin and mRFP-VP26 capsid fluorescence, and L-particles (light particles), categorized based on the absence of mRFP-VP26 (Fig 1C and 1D). With this PRV mutant at this time point, we observed approximately two thirds of particles undergoing exocytosis are L-particles, and one third are virions (Fig 1C). Previously, we observed that exocytosis of PRV virions occurs as early as 4.5–5 hours post-infection (hpi) in non-neuronal PK15 cells [19]. Here we observed PRV particles (virions or L-particles) undergoing exocytosis from the cell body of SCG neurons as early as 5–6 hpi, much earlier than the previously-reported increases in neuronal activity and increases in intracellular Ca^2+^ at 8–10 hpi [11,31].

### PRV particle egress from neuronal cell bodies uses Rab6a and Rab8a-positive secretory vesicles

To characterize the secretory organelle used for PRV egress from neuronal cell bodies, we first expressed mCherry-tagged Rab GTPases in primary SCG neurons using non-replicating adenovirus vectors. Approximately 18 hr after vector transduction, we infected with PRV 486, expressing gM-pHluorin, and imaged cell bodies by TIRF microscopy to detect viral exocytosis events at 5–6 hr after PRV infection. To generalize the relationship between fluorescent signals, we measured gM-pHluorin and mCherry-Rab fluorescence of many exocytosis events, aligned them based on moment of exocytosis, and calculated an ensemble average of relative fluorescence intensity over time (Fig 1E and 1F). An increase in Rab fluorescence before exocytosis represents the gradual arrival of Rab-positive secretory vesicles to the site of exocytosis. After exocytosis, Rab protein fluorescence rapidly decays, representing diffusion of Rab proteins away from the site of exocytosis upon GTP hydrolysis (Fig 1E and 1F).

Using this method, we found that Rab6a and Rab8a are dynamically associated with exocytosis of virus particles (virions or L-particles) from cell bodies (Fig 1E and 1F), in agreement with our previous results in non-neuronal cells. In contrast, we previously observed that Rab11a, which has many functions including trafficking of recycling endosomes, is also associated with viral secretory vesicles in non-neuronal cells [19,20]. However, we show here that Rab11a does not appear to be associated with virus particle exocytosis from the cell body of neurons (Fig 2A), consistent with the divergent roles of Rab11 proteins in neurons versus non-neuronal cells [26]. Finally, Rab3a, which mediates the trafficking of synaptic vesicle precursors and Ca^2+^-regulated exocytosis of synaptic vesicles in neurons, also does not appear to be associated with virus particle exocytosis from SCG cell bodies (Fig 2B).

### PRV particle exocytosis from neuronal cell bodies is not correlated with local Ca^2+^ dynamics

In addition to global membrane depolarization and Ca^2+^ transients associated with action potentials, neurons and other excitable cell types, including SCG neurons in particular [32], also exhibit smaller localized Ca^2+^ transients, variously described as “Ca^2+^ sparks”, “Ca^2+^ glows”, “syntillas”, or “Ca^2+^ microdomains”. These local Ca^2+^ dynamics are highly variable in amplitude, time course, and likely result from a variety of intracellular signaling pathways [32–36]. To determine whether virus exocytosis events are correlated with local Ca^2+^ dynamics in cell bodies, we infected primary SCG neurons with PRV 486, expressing gM-pHluorin, loaded neurons with the membrane permeant Ca^2+^-sensitive dye Rhod-2-AM, and imaged beginning at 5 hpi. Infected neurons exhibited local Ca^2+^ dynamics indicated by local changes in Rhod-2-AM fluorescence intensity (Fig 2C and 2D). To generalize the relationship between fluorescent signals, we measured the gM-pHluorin and Rhod-2-AM fluorescence of many exocytosis events, aligned them to a common timescale based on the moment of exocytosis, and calculated an ensemble average. In this analysis, exocytosis of virus particles (virions or L-particles) was not associated spatially or temporally with local Ca^2+^ dynamics (Fig 2E), consistent with previous studies indicating that individual local Ca^2+^ transients are not correlated with spontaneous exocytosis in uninfected neurons and neuroendocrine cells [34,35].

### PRV particles can colocalize with Rab6a in the proximal axon, but co-transport is not detected in distal axons

To investigate whether the Rab6 secretory pathway mediates axonal sorting and transport of PRV particles, we infected primary SCG neurons with PRV IH222, expressing EmGFP-Rab6a and mCherry-VP26 capsid protein. Neurons were cultured either on glass coverslips to image cell bodies and proximal axons at 5–10 hpi, or in modified Campenot tri-chambers to image distal axons at 10–24 hpi.

**Fig 3:**
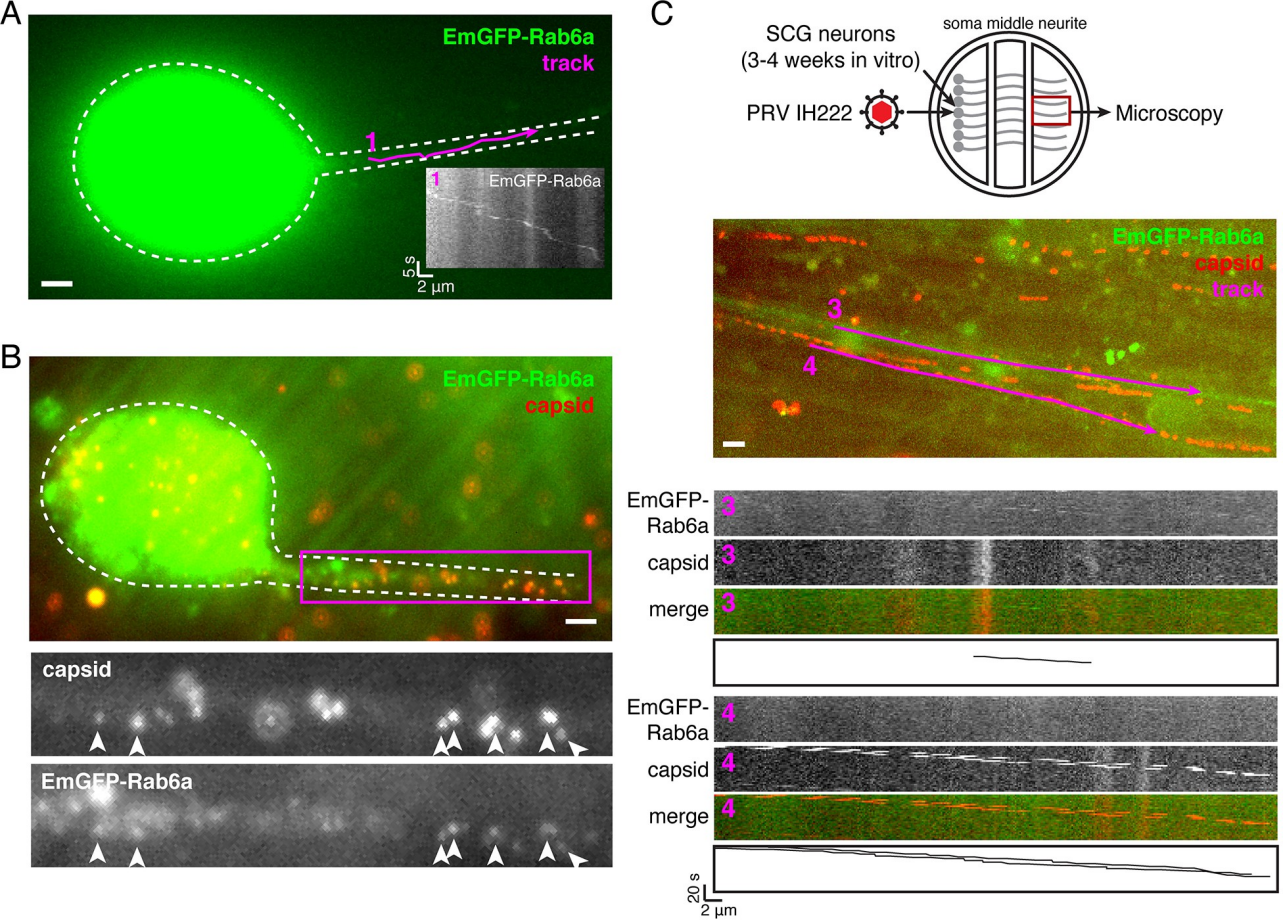
Some PRV particles colocalize with Rab6a in the proximal axon, but Rab6a is not detected with PRV particles in distal axons. (A-B) SCG neurons were infected with PRV expressing mCherry-VP26 and EmGFP-Rab6a, and imaged beginning at 5 hr post-infection. Data are representative of 50 cells over 7 independent experiments. Scale bars represent 4 μm. (A) EmGFP-Rab6a vesicles undergo sorting and transport into the proximal axon. Kymograph (inset) represents particle movement along the indicated track (magenta). (B) PRV particles (mCherry-VP26) colocalize with EmGFP-Rab6a in the proximal axon (arrowheads). (C) EmGFP-Rab6a vesicles transport to the distal axon (top set of kymographs), but EmGFP-Rab6a is not detectable on PRV particles (bottom kymographs). Kymographs represent particle movement along the indicated tracks (magenta). Data are representative of ~500 capsid tracks, from 36 independent fields of view, over 4 independent experiments. Scale bar represents 4 μm.

In the neuronal cell biological literature, Rab6 is used as a marker of secretory and dense core vesicles in axons, and mediates vesicular transport to synapses in particular [37–39]. Consistent with these reports, we readily detected transport of EmGFP-Rab6a puncta in the proximal (Fig 3A) and distal (Fig 3C) axons at all time points.

Some PRV particles in proximal axons colocalize with EmGFP-Rab6a (Fig 3B). We acquired 50 images of infected SCG neurons, focusing on the proximal axons, over 7 independent experiments. In essentially all proximal axons, we could detect PRV particles that colocalized with EmGFP-Rab6a, but many individual particles had very low or undetectable EmGFP-Rab6a fluorescence.

To determine whether PRV particles are associated with Rab6 during long-distance transport in distal axons, we cultured SCG neurons in Campenot tri-chambers, which fluidically separate SCG cell bodies from their axons [36,37]. Primary SCG neurons were seeded into the left soma chamber. After approximately 2 weeks in culture, SCG neurons extend axons under the chamber walls into the rightmost neurite chamber. This compartmented culture system allows us to infect neuronal cell bodies, but the fluidically-separated axons are not directly exposed to viral inoculum. Thus, any virus particles observed in the distal axons following viral replication represent viral progeny, not inoculum.

Following axon extension into the right neurite chamber (3–4 weeks in vitro), we infected with PRV IH222 in the left soma compartment, and imaged virus particle transport in distal axons in the right neurite compartment at times ranging from 10–24 hpi. In these tri-chamber cultures, many axons extend into the right neurite chamber; thus, detecting transport of PRV particles in distal axons is very frequent because we can visualize many distal axons in a single field of view. We imaged >500 particles transporting in axons, across 36 fields of view each containing hundreds of axons, over 4 independent experiments. We readily detected EmGFP-Rab6a vesicles moving in distal axons (Fig 3C), indicating that these secretory organelles are capable of long-distance axonal transport. We also readily detected mCherry-VP26 PRV particles transporting anterograde in distal axons. However, we were unable to detect any colocalization or co-transport (Fig 3C).

Altogether, these results show that Rab6 vesicles mediate egress of PRV from the cell body, and might contribute to sorting of PRV particles into the proximal axon, but Rab6a does not appear to contribute to longer-distance transport of PRV particles to distal axons. It remains to be determined whether Rab6 is deactivated during axonal sorting and transport, or if there is a different population of vesicles that mediate axonal egress of PRV.

### Investigating the relationship between neuronal activity and axonal egress and spread

Because the specific secretory factors used by PRV for egress from distal axons remains unclear, we instead adopted a more general approach to determine whether axonal egress uses Ca^2+^-regulated secretory mechanisms, dependent on neuronal activity, versus constitutive secretory mechanisms that do not depend on neuronal activity.

In primary SCG neurons *in vitro*, PRV causes a significant increase in firing activity by 8–10 hours post-infection (hpi) [11], and a significant increase in intracellular Ca^2+^ accumulation [31]. While we demonstrated above that viral egress from the cell body occurs as early as 5 hpi (before detectable increases in neuronal activity), at a typical kinesin motor velocity of 1–3 μm/s, it would take an additional 1–3 hr for the earliest progeny virus particles reach the distal axons in the Campenot tri-chamber (~1 cm). Therefore, axonal egress of PRV particles could coincide with the reported increases in neuronal activity.

**Fig 4:**
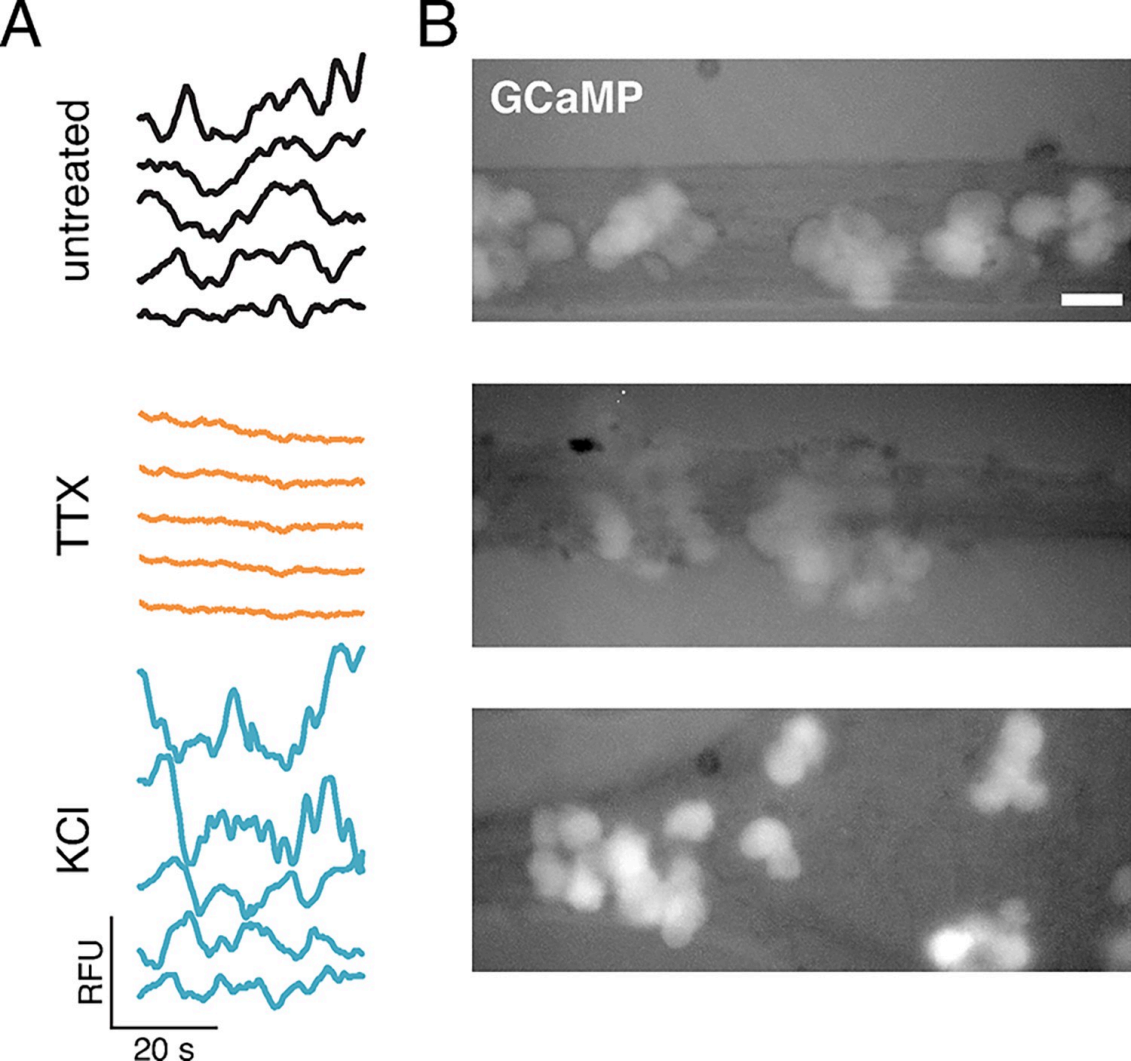
Tetrodotoxin and KCl affect activity of infected neurons. Primary SCG neurons in modified Campenot trichambers were infected with a recombinant PRV expressing the fluorescent Ca^2+^ sensor GCaMP3, and treated with 4μM tetrodotoxin (TTX), pulses of 55mM KCl, or untreated, as indicated. (A) Relative Ca^2+^-dependent GCaMP3 fluorescence of 5 representative cells over time. Relative fluorescence units (RFU) are scaled identically between experimental conditions. (B) Steady-state Ca^2+^ accumulation indicated by GCaMP3 fluorescence. Scale bar represents 40 μm.

To modulate neuronal firing activity, we first used tetrodotoxin (TTX), which prevents neuronal firing by blocking voltage-gated Na^+^ channels, and potassium chloride (KCl), which directly excites neurons by depolarizing their membrane potential. To ensure that TTX and KCl function in the context of PRV infection and in Campenot tri-chamber cultures, we infected primary SCG neurons with PRV 468, expressing the fluorescent protein Ca^2+^ sensor GCaMP3, and monitored their Ca^2+^-dependent fluorescence between 9–11 hpi, in the presence or absence of these drugs. GCaMP3 cannot resolve individual action potentials, but GCaMP fluorescence instead represents a running average of recent cytosolic Ca^2+^ concentration. TTX (4 μM) was added beginning at 4 hpi, and KCl (55 mM) was perfused in pulses beginning at 6 hpi. Untreated neurons exhibited an intermediate variation in Ca^2+^-dependent fluorescence over time (Fig 4A), and an intermediate steady-state accumulation of Ca^2+^-dependent fluorescence (Fig 4B). TTX treatment effectively silenced neuronal activity and reduced steady-state Ca^2+^ accumulation. All cells were responsive to KCl treatment, exhibiting a higher steady-state accumulation of Ca^2+^-dependent GCaMP3 fluorescence (Fig 4B), but exhibited cell-to-cell variability in the variance of the fluorescence over time (Fig 4A). These cell-to-cell differences are likely due to saturation of GCaMP3 at very high, non-physiological cytosolic Ca^2+^ concentrations. These data indicate that TTX and KCl are effective during PRV infection, and under our experimental conditions.

### Neuronal activity does not promote PRV spread from neuronal axons to non-neuronal cells

**Fig 5:**
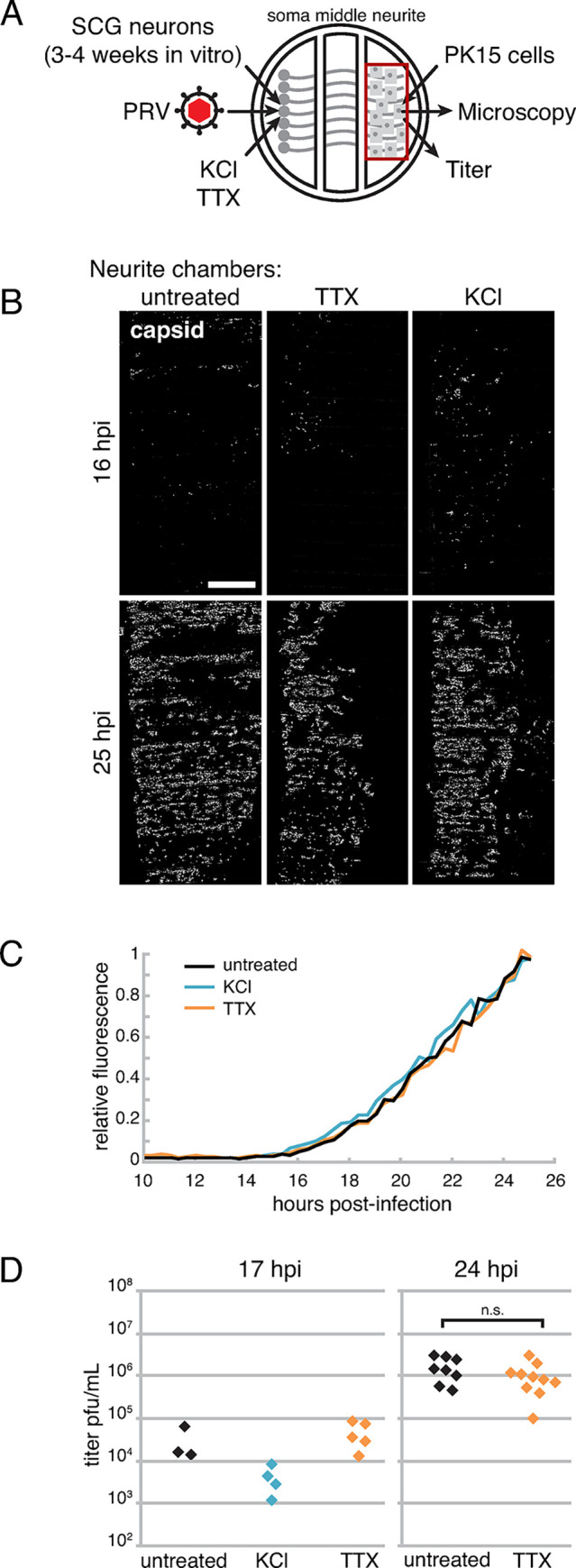
Neuronal activity does not correlate with PRV spread from neurons to non-neuronal cells. (A) Schematic of modified Campenot tri-chamber. SCG neurons were seeded in the left soma compartment and extended axons under the chamber walls to the right neurite compartment. A detector cell monolayer (PK15) was then added to the neurite compartment. PRV infection was initiated in the soma compartment, and neurons were treated with 4μM TTX, or pulses of 55mM KCl, or untreated, as indicated. (B) Tiled image of neurite chambers (area indicated by red box in panel A). Scale bar represents 1mm. (C) Relative mRFP-VP26 capsid fluorescence of entire neurite chambers over time. (D) Virus titer measured in neurite chambers by serial dilution plaque assay.

To determine whether neuronal activity affects axonal egress and spread of PRV to non-neuronal recipient cells, we first cultured SCG neurons in the left soma compartment of Campenot tri-chambers for 3 weeks, to allow axon extension into the right neurite compartment. After 3 weeks in vitro, we then seeded non-neuronal PK15 recipient cells onto the axons in the right neurite chamber (Fig 5A). Neurons in the left soma chamber were infected with either PRV Becker, or PRV 180 that expresses an mRFP-VP26 capsid tag, and treated with TTX (4 μM) or KCl (55 mM) from 1 hpi. We monitored the extent of virus spread to recipient PK15 cells by live-cell fluorescence microscopy, acquiring large tiled images of the entire neurite compartment every 20 min., beginning at 10 hpi (Fig 5B and 5C). We also performed plaque assays measuring virus titer in the neurite compartment (Fig 5D). The first mRFP-VP26 expression was detected in the recipient PK15 cells by 16 hpi in all conditions (Fig 5B), and we did not observe any differences in the kinetics of spread over time (Fig 5C). TTX treatment had no effect on virus titer in the neurite compartment at 17 or 24 hpi, and KCl treatment caused an ~1 log reduction in neurite compartment titer at 17 hpi (Fig 5D).

### Neuronal activity does not promote PRV spread from neuron to neuron

To determine whether neuronal activity affects spread of PRV from neuron to neuron, we prepared tri-chamber cultures with SCG neurons as recipient cells.

**Fig 6:**
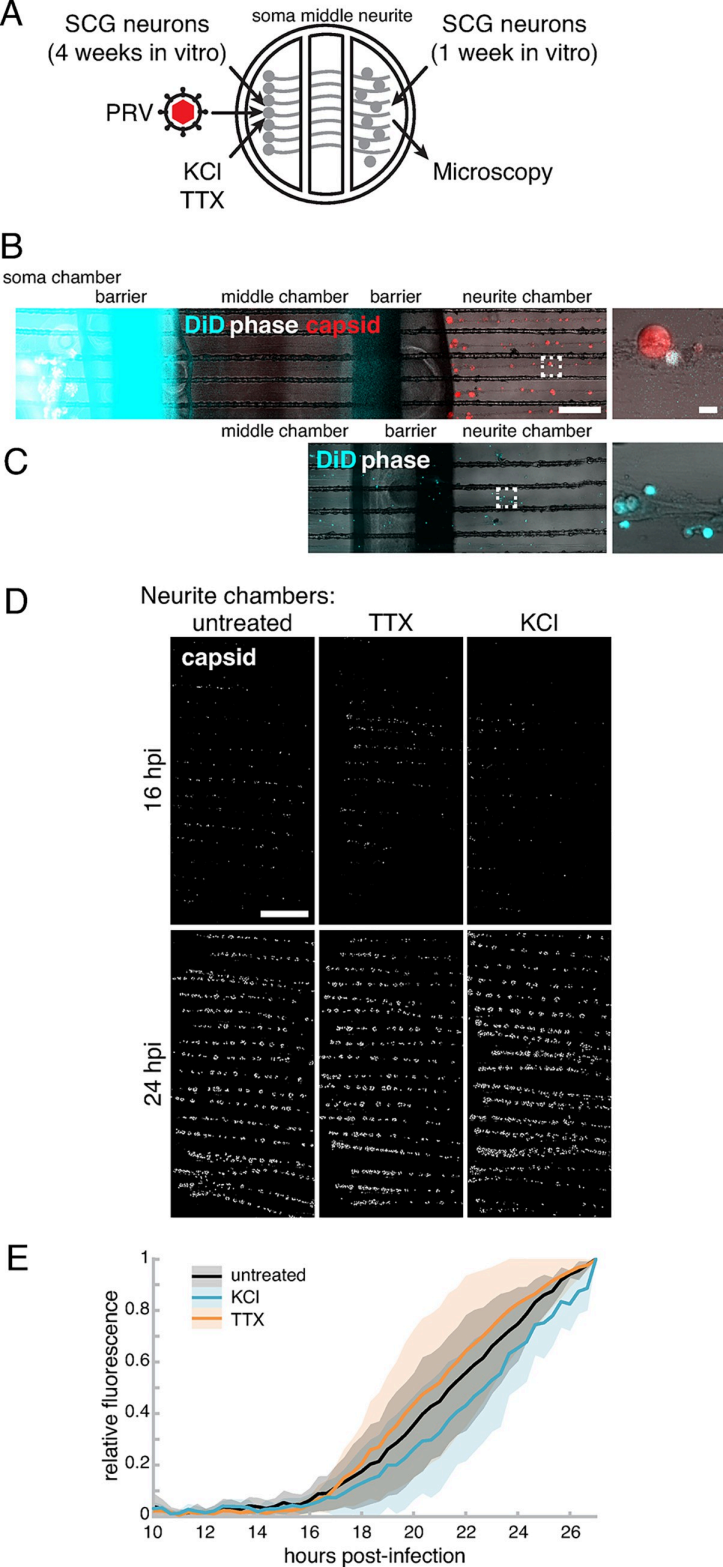
Neuronal activity does not correlate with PRV spread from neuron to neuron. (A) Schematic of modified Campenot tri-chamber. SCG neurons were seeded in the left soma compartment and extended axons under the chamber walls to the right neurite compartment. Recipient SCG neurons were then added to the neurite compartment. PRV infection was initiated in the soma compartment, and neurons were treated with 4μM TTX, or pulses of 55mM KCl, or untreated, as indicated. (B) Recipient neurons in the right neurite chamber do not penetrate the left soma compartment. Lipophilic fluorescent dye, DiD (blue), was added to the soma chamber, and chambered cultures were imaged by live-cell fluorescence microscopy. Out of many chambers, only a single DiD-labeled cell body was detected (zoom). Scale bars represent 0.5 mm (left) and 30 μm (zoom). (C) A defective leaky chamber demonstrates extensive DiD-labeling of cells, demonstrating that these imaging parameters can readily detect DiD-positive cells in the neurite chamber. (D) Tiled image of neurite chambers (area indicated by red box in Fig 2A). Scale bar represents 1mm. (E) Mean relative mRFP-VP26 capsid fluorescence of entire neurite chambers over time. Mean of 8 independent experiments. Shaded area represents standard deviation. No significant difference (p>0.05) at all time points.

We first cultured SCG neurons in the left soma compartment of Campenot tri-chambers for 3 weeks, to allow axon extension into the right neurite compartment. We then seeded additional freshly-prepared SCG neurons onto the axons in the neurite compartment and maintained these cultures for an additional 1 week (Fig 6A). The Banker laboratory previously described such “heterochronic cultures” (i.e. co-culture of neurons of different ages) and observed synapses forming between hippocampal neurons within 1–3 days [40]. It is well established that dissociated SCG neurons form functional adrenergic and cholinergic synapses in vitro (discussed in [41,42]), and synaptogenesis can be detected within 6–7 days in vitro [43–45].

To ensure that newly-added recipient SCG neurons cannot extend axons though the chamber barriers into the left soma chamber within 1 week, we added the lipophilic far-red fluorescent dye DiD to the left soma chamber (Fig 6B and 6C). Any recipient SCG neurons that had extended axons into the soma chamber would become labeled by the fluorescent dye. We acquired large tiled images of entire tri-chamber cultures by fluorescence microscopy, and observed only one instance of a single DiD-labeled recipient neuron in the neurite chamber (Fig 6B). As a control, a defective tri-chamber culture that leaked DiD into the middle compartment had a much greater amount of DiD labeling in recipient neurons, demonstrating that our imaging parameters can readily detect DiD-positive cells (this defective chamber was not used for any subsequent experiments) (Fig 6C).

Neurons in the left soma chamber were infected with PRV 180, expressing the mRFP-VP26 capsid tag, then TTX (4 μM) was added beginning at 5 hpi, or KCl (55 mM) was perfused in pulses beginning at 6 hpi (Fig 6A). The extent of virus spread to recipient neurons in the neurite chamber was monitored by fluorescence microscopy, acquiring large tiled images of the entire neurite compartment every 20 min., beginning at 10 hpi. In all conditions, the first mRFP-VP26 expression was detected by 16 hpi (Fig 6D), and we did not observe any significant (p>0.05) differences in the kinetics of spread over time (Fig 6E).

### Neuronal activity does not promote PRV spread from neuronal axons: Optogenetics Approach

**Fig 7:**
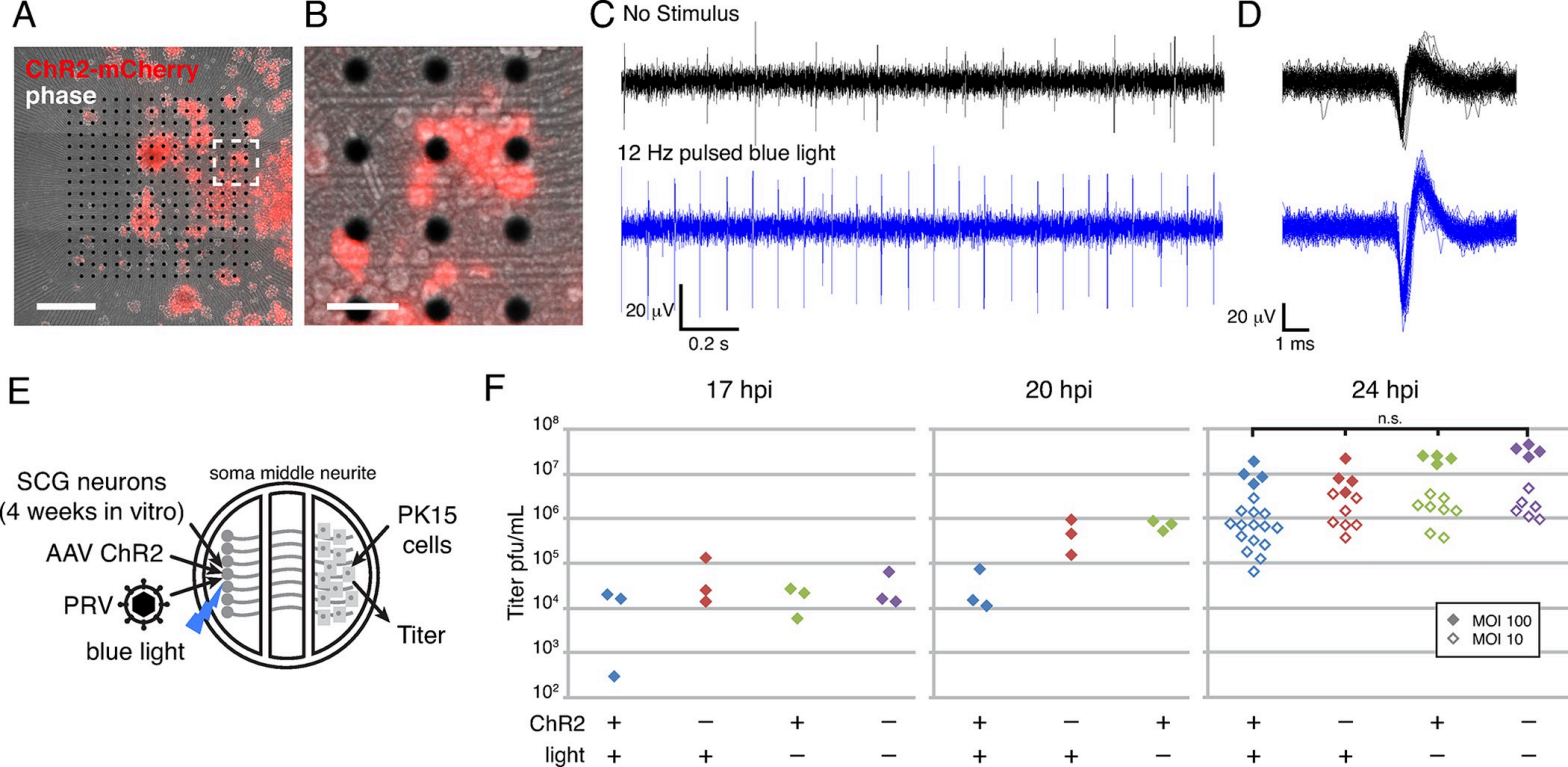
Neuronal activity does not correlate with PRV spread from neurons: optogenetics approach. (A-B) SCG neurons were seeded onto a multi-electrode array, transduced with an AAV vector expressing ChR2-mCherry. (A) Scale bar is 500μm. (B) Scale bar is 100μm. (C) Extracellular electrical recordings of SCG neurons exhibiting spontaneous spiking activity (no stimulus), or evoked activity synchronized to 12 Hz blue light pulses. (D) Superimposed spikes (n = 100) demonstrating increases in spike peak-to-peak amplitude with optogenetic stimulation. (E) Schematic of modified Campenot tri-chamber. SCG neurons were seeded in the left soma compartment and extended axons under the chamber walls to the right neurite compartment. A detector cell monolayer (PK15) was then added to the neurite compartment. SCGs were transduced with a ChR2 AAV and exposed to pulsed blue light, as indicated. PRV infection was initiated in the soma compartment at a multiplicity of infection of 10 or 100, as indicated. (F) Virus titer measured in neurite chambers by serial dilution plaque assay. No significant difference (p>0.05) at 24 hpi.

Because KCl treatment causes excitotoxicity in neurons and has been previously reported to reduce HSV-1 replication [46], we next pursued an optogenetics approach to modulate neuronal firing activity. Channelrhodopsin 2 (ChR2) is a light-gated ion channel that allows control of neuronal activity using light [47]. To validate this method, we first plated SCG neurons on a multi-electrode array, transduced these neurons with an AAV vector to express ChR2-mCherry (Fig 7A and 7B), and performed extracellular recordings of their spontaneous and light-evoked spiking activity (Fig 7C). Greater than 80% of neuronal cell bodies expressed ChR2-mCherry red fluorescence (Fig 7A and 7B). Without blue light stimulus, neurons exhibited an average spontaneous spiking frequency of 4 Hz. Neurons were then exposed to blue light pulsed at approximately 12 Hz. With blue light stimulation, neuron activity was synchronized to the pulsed blue light, with a measured average spiking frequency of ~11 Hz. Light stimulation also increased peak-to-peak spike amplitude from an average of 70 μV to 108 μV (Fig 7C and 7D).

To determine whether optogentically-controlled neuronal activity affects spread of PRV, we prepared tri-chambers with SCG neurons in the left soma chamber, and transduced with an AAV vector to express ChR2-mCherry. We screened neurons by fluorescence microscopy to ensure ChR2-mCherry expression in >80% of cells. At 4 weeks in vitro, we seeded PK15 recipient cells in the right neurite compartment, infected with PRV Becker, and pulsed with blue light beginning at 1 hpi (Fig 7E). In the experimental condition, where neurons were transduced with ChR2 and pulsed with blue light, neurite compartment titers were slightly less than controls at 17 and 20 hpi, but not significantly different by 24 hpi (p>0.05) (Fig 7F).

Altogether, these results show that silencing neuronal activity does not inhibit, and artificially elevating neuronal activity does not increase PRV egress and spread from axons. Thus, while the specific secretory mechanisms used by alpha herpesviruses to exit from axons remains elusive, we conclude that elevated neuronal activity, and therefore activity-dependent Ca^2+^-regulated secretory mechanisms, are not necessary for efficient viral egress and spread from neurons.

## Discussion

Based on three main lines of evidence, 1) that alpha herpesviruses spread in a synaptic circuit-specific manner in the mammalian nervous system; 2) co-localization between alpha herpesvirus proteins/particles and some synaptic vesicle markers has been reported; and, 3) that alpha herpesviruses cause an elevated rate of neuronal activity *in vitro* and *in vivo*, it has long been suspected that alpha herpesviruses take advantage of synaptic vesicle-like regulated secretory mechanisms in neurons.

Here we present several lines of evidence that contradict this view. Using live-cell fluorescence microscopy, we show that PRV particles colocalize and co-transport with Rab6a in the cell body, and that PRV particle exocytosis from the cell body is associated with Rab6a and Rab8a. We observed no association between virus particle exocytosis and intracellular Ca^2+^ signaling, and no association with Rab3, which regulates the intracellular trafficking and Ca^2+^-dependent exocytosis of synaptic vesicles. Although some PRV particles colocalize with Rab6 in the proximal axon, we did not observe any colocalization or co-transport with Rab6a in distal axons. It remains unclear whether Rab6 is initially present on all viral particles and is subsequently deactivated during axonal sorting and transport, or if there is a distinct population of Rab6-negative organelles that mediate viral axonal sorting and transport. Since another study detected Rab3a on some enveloped virions in distal axons [27], it is possible that these account for the Rab6a-negative particles we observe.

While the specific cellular factors used in distal axons remain uncertain, using Campenot tri-chambers to measure axonal egress and spread, and tetrodotoxin to silence neuronal activity, we observed no inhibition of virus spread from axons. Using KCl or optogenetics to elevate neuronal activity, we also show no increase in virus spread from axons. Therefore, we conclude that PRV egress and spread is associated with constitutive secretory mechanisms in the neuron cell body, and egress from axons is also independent of neuronal activity.

There are several important limitations to this study. Foremost, while SCG neurons do form functional synapses *in vitro*, measuring transneuronal virus spread through the Campenot tri-chamber cannot distinguish between spread via these neuronal synapses and spread via other non-synaptic cell-cell contacts. We note that, in this study, we did not attempt to visualize neuronal synapses or axonal exocytosis, and we were unable to detect Rab6 trafficking with virus particles in distal axons. It is therefore possible that alpha herpesviruses use constitutive secretory mechanisms for egress from the cell body and other non-synaptic axonal egress sites, but also use Ca^2+^-regulated secretory mechanisms for egress specifically at neuronal synapses. To resolve these remaining ambiguities, in future studies it will be necessary to directly observe virus egress and spread specifically at neuronal synapses to distinguish the molecular and cellular mechanisms of synaptic versus non-synaptic cell-cell spread.

## Materials and methods

### Cells

PK15 cells (ATCC CCL-33) were cultured in Dulbecco’s Modified Eagle Medium (DMEM) supplemented with 10% fetal bovine serum (FBS) and 1% penicillin/streptomycin, at 37°C and 5% CO_2_. Primary embryonic rat superior cervical ganglion (SCG) neurons were isolated and cultured as previously described [36,37]. To measure anterograde spread, neurons were cultured in modified Campenot tri-chambers, as previously described [46,47]. For TIRF microscopy, SCG neurons were cultured on 35mm glass-bottom tissue culture dishes (Mattek, Ibidi, or Celltreat), which were treated by corona discharge (Electro-Technic Products) and sequentially coating with poly-DL-ornithine and laminin. For extracellular electrode recording, SCG neurons were cultured on a multielectrode array (Multichannel Systems), which was prepared by sequentially coating with polyethylenimine (PEI) and laminin. In all cases, neurons were cultured in Neurobasal medium supplemented with B-27 (ThermoFisher), glutamine, penicillin/streptomycin, and nerve growth factor (NGF), for 2–4 weeks to achieve a fully-differentiated and polarized neuronal phenotype.

### Ethics statement

Animals were euthanized by carbon dioxide inhalation, as recommended by the American Veterinary Medical Association (AVMA). All animal work was approved by the Institutional Animal Care and Use Committees at Princeton University (protocol #1947) or Arizona State University (protocol #20-1799R & 23-2011R), in accordance with applicable policies and laws, including the Animal Welfare Act (AWA), the Public Health Service Policy on Humane Care and Use of Laboratory Animals, the Principles for the Utilization and Care of Vertebrate Animals Used in Testing, Research and Training, and the Health Research Extension Act of 1985.

### Viruses

All PRV recombinants are derivatives of PRV Becker, a non-syncytial wild-type laboratory strain [48]. PRV 180 and PRV 960, which express mRFP-VP26 or mCherry-VP26 capsid protein fusions, respectively, were previously described [49]. PRV 468, expressing mRFP-VP26 and the fluorescent protein Ca^2+^ sensor GCaMP3, was previously described [12]. PRV 486, expressing mRFP-VP26 and gM-pHluorin, was previously described [19]. PRV 616, which contains a CMV promoter, mRFP coding sequence, and SV40 polyadenylation signal inserted into the US4/gG locus, was previously described [50].

PRV IH222 was constructed as follows: Plasmid pCPD-HSV-N-EmGFP-Rab6a was produced by the DNASU plasmid repository (Arizona State University) and expresses Emerald GFP (EmGFP) fused to the N-terminus of Rab6a (GenBank EU832786.1). The fusion junction between EmGFP and Rab6a is as follows: …GMDELYK**GSSPSTSLYKKAGST***MSTGGDFGNP*… (the C-terminal sequence of EmGFP is shown in plain font, a 15 peptide linker is in boldface, and the N-terminal sequence of Rab6a is italicized).

To produce PRV IH003, PK15 cells were co-transfected with PRV 616 genomic DNA and linearized pCPD-HSV-N-EmGFP-Rab6a. Following reconstitution of replicating virus, recombinant plaques were selected based on loss of mRFP and gain of EmGFP-Rab6a fluorescence. Finally, PRV IH222 was constructed by coinfecting PRV IH003 and PRV 960 and screening for two-color plaques expressing both EmGFP-Rab6a and mCherry-VP26.

SCG neurons were infected with PRV recombinants at a multiplicity of infection (MOI) of approximately 5 plaque-forming units (pfu)/cell, unless stated otherwise in the figure or figure legend. Since low MOI infection can produce variability in early stages of infection, we infect at a high MOI to ensure a robust and synchronous lytic replication, to be able to reliably measure egress/cell-cell spread late in infection. Virus concentration was measured by serial dilution plaque assay, as follows: cells and supernatants were harvested by scraping, freeze-thawed, sonicated, and serially diluted. Dilutions were inoculated onto PK15 cell monolayers, overlaid with cell culture medium thickened with 2% methocel (Dupont), and incubated until plaques are visible to the naked eye.

Adenovirus vectors expressing mCherry-tagged Rab3a, Rab6a, Rab8a, and Rab11a were previously described [19]. SCG neurons were transduced with adenovirus vectors approximately 18 hours before co-infection with PRV. An adeno-associated virus (AAV) vector expressing channelrhodopsin2 (ChR2) fused to mCherry (AAV2/1.CAG.hChR2(H134R)-mCherry.WPRE.SV40) was obtained from the U. Pennsylvania Vector Core. SCG cultures were inoculated with 10^7^ transducing units of AAV and screened by fluorescence microscopy for >80% transduction efficiency.

### Optogenetics and extracellular electrode recording

SCG neurons transduced with AAV vectors expressing ChR2 were exposed using a blue LED light box with an excitation spectrum of approximately 457/46 nm (Clare Chemical Research, Denver, CO, USA) [51], driven by an Arduino Uno Rev3 microcontroller programmed to deliver light pulses at approximately 12 Hz (see S1 Text). Extracellular recordings of spontaneous and light-evoked spiking activity were digitized at 10 kHz and stored for offline analysis, as previously described [52]. The extracellular recordings were composed of fast negative voltage spikes from individual neurons, superimposed on a slowly fluctuating local field potential generated by the average activity of many nearby neurons. Recordings were processed by subtracting electronic noise recorded on a reference electrode and 200 Hz high-pass filtered to remove local field potentials, spikes were identified using a threshold of -30 μV, and average spike frequency and peak-to-peak amplitude were measured using MC_Rack software (Multichannel Systems).

### Fluorescence microscopy and image processing

Tiled images of entire neurite compartments in tri-chambers were acquired using an automated epifluorescence microscope equipped with a 37°C and 5% CO_2_ environmental chamber for long-term imaging of live cells, as previously described [53]. To measure the total fluorescence in chambered neuronal cultures, we performed background subtraction and feature selection using median filter background subtraction and granulometric filtering in Fiji/ImageJ v.1.52b-2.9.0/1.53t software, as previously described [54]. TIRF microscopy was performed using a Nikon N-STORM fluorescence microscope in the Princeton University Confocal Imaging Facility, as previously described [19,20], or a Nikon Eclipse Ti2-E fluorescence microscope, equipped with a TIRF illuminator, 488nm and 561nm lasers, a Photometrics Prime95B sCMOS camera, a 60X high-NA TIRF objective, and objective and stage warmers for 37°C live-cell microscopy, in the Biodesign microscopy core facility at Arizona State University. Images were prepared for publication using the following functions and plugins in Fiji/ImageJ: adjust brightness and contrast, Z project (to make maximum intensity projections), Reslice (to produce kymographs), and Plot Z-axis Profile (to measure fluorescence over time). We calculated maximum difference projections in Fiji/ImageJ as follows: Image values at time n were subtracted from values at time n+5 to identify pixels where fluorescence intensity increases, and the resulting image stacks were then processed by maximum intensity projection to highlight areas where fluorescence intensity increases the most.

The numerical data for bar, line, and scatter plots in figures are included in S1 Data.

## Supporting information

S1 Data Excel spreadsheet containing, in separate sheets, the underlying numerical data for Figs 1A, 1D, 1E, 1F, 2A, 2B, 2E, 5C, 5D, 6E, and 7E. (XLSX)

S1 Text Supporting Materials and Methods: Arduino Controller for Optogenetics Light Stimulation. (PDF)
