# Supplementary material for: Alpha herpesvirus exocytosis from neuron cell bodies uses constitutive secretory mechanisms, and egress and spread from axons is independent of neuronal firing activity
Source: PLoS Pathog. 2024 Apr 5;20(4):e1012139. doi: 10.1371/journal.ppat.1012139 (PMC11023632; doi:10.1371/journal.ppat.1012139)
Supplement: S1 Text — (PDF) [file ppat.1012139.s002.pdf]

Supporting Information

Alpha Herpesvirus Exocytosis from Neuron Cell Bodies Uses Constitutive Secretory Mechanisms, and Egress and Spread from Axons is Independent of Neuronal Firing Activity

Anthony E. Ambrosini, Kayla M. Borg, Nikhil Deshmukh, Michael J. Berry II, Lynn W. Enquist, Ian B. Hogue

S1 Supporting Materials and Methods.

Arduino Controller for Optogenetics Light Stimulation

An Arduino controller (Arduino Uno R3, Arduino, Milan, Italy) was built and programmed to control of a relay switch (PowerSSR Tail, powerswitchtail.com, Honolulu, HI), timing the on/off cycle of a transilluminator (Clare Chemical Research, Dolores, CO) to drive channelrhodopsin-2 activity in long-term optogenetics experiments inside a cell culture incubator.

Two dial potentiometers control the on rate and off rate at frequencies up to 30 Hz. Due to response delay in the lightbox, the reported on and off times should be treated as a reference only (reported on time is an overestimate and off time is an underestimate). Measurements should be made with a photosensor if more precise on/off rate is sought.

Below is the code for the Arduino controller that is designed to interact with circuitry illustrated in Figure S1. The code is written in the Arduino programming language, which is derivative of C/C++.

```
#include <LiquidCrystal.h>
LiquidCrystal lcd(12, 11, 5, 4, 3, 2);
const int ledPin =    9;
const int switchPin = 6;
const int pot1Pin = 0;
const int pot2Pin = 2;
int on_delay = 300; int off_delay = 700;
int power_state;
long int timer1; long int timer2;

void setup()
{
  pinMode(switchPin, INPUT);
  pinMode(ledPin, OUTPUT);
  Serial.begin(9600);
  digitalWrite(ledPin, LOW);
  lcd.begin(16, 2);
  lcd.print(" on time: ");
  lcd.setCursor(0, 1);
  lcd.print("off time: ");
}

void loop()
{
  on_delay = max(analogRead(pot1Pin)/2,25);
  off_delay = analogRead(pot2Pin);
  power_state = digitalRead(switchPin);
  Serial.print("power: ");
  Serial.print(power_state);
  Serial.print(";   on time: ");
  lcd.setCursor(10, 0);
  lcd.print(on_delay);
  lcd.print("ms ");
  Serial.print(on_delay);
  Serial.print("ms;   off time: ");
  Serial.print(off_delay);
  Serial.println("ms");
  lcd.setCursor(10, 1);
  lcd.print(off_delay);
  lcd.print("ms ");
  if(power_state)
  {
    timer1 = millis();
    digitalWrite(ledPin, HIGH);
    delay(on_delay);
    timer2 = millis();
    digitalWrite(ledPin, LOW);
```

```
    Serial.print("             actual: ");
    Serial.print(millis()-timer1);
    delay(off_delay);
    Serial.print("ms      actual: ");
    Serial.print(millis()-timer2);
    Serial.print("ms\r\n\r\n");
  }
  else
  {
    delay(1000);
  }
}
```

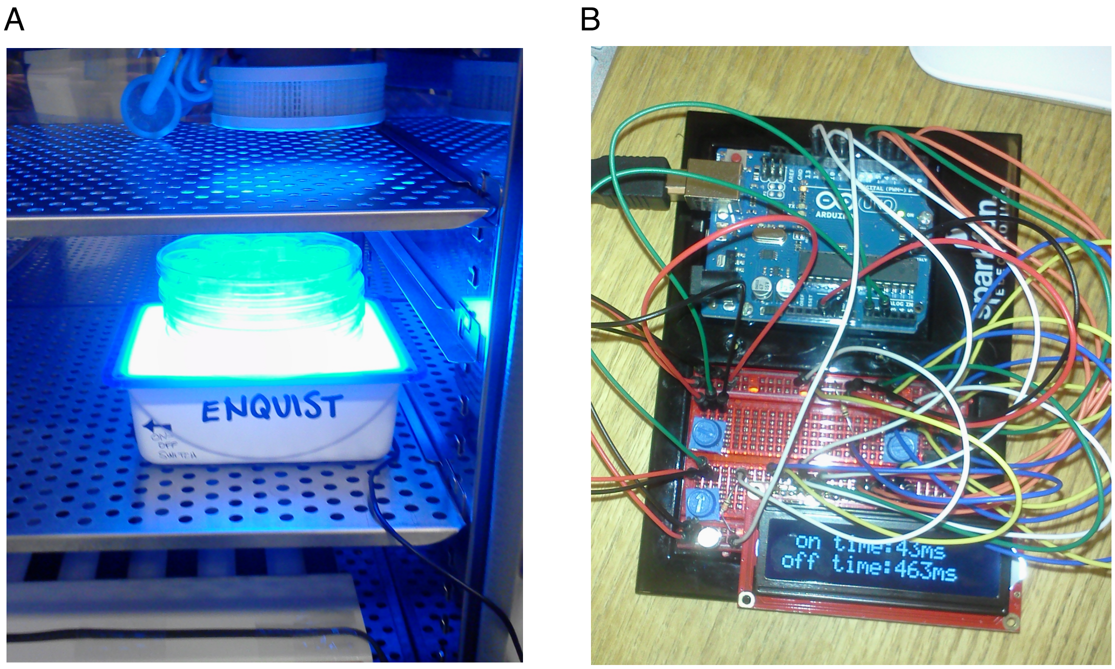

S1 Figure. Arduino microcontroller circuitry for optogenetics light stimulation. (A) Photograph of blue light transilluminator with cell culture dishes installed in a cell culture incubator. (B) Photograph of Arduino microcontroller with wiring (arbitrary wire colors).
